# Droplet digital PCR using HER2/EIF2C1 ratio for detection of HER2 amplification in breast cancer tissues

**DOI:** 10.1007/s12032-018-1210-8

**Published:** 2018-10-03

**Authors:** Anchalee Tantiwetrueangdet, Ravat Panvichian, Sansanee Wongwaisayawan, Natthaporn Sueangoen, Panuwat Lertsithichai

**Affiliations:** 1 Research Center, Faculty of Medicine, Ramathibodi Hospital, Mahidol University, Bangkok, Thailand; 2 Department of Internal Medicine, Division of Medical Oncology, Faculty of Medicine, Ramathibodi Hospital, Mahidol University, Rama 6 Road, Rajthevi, Bangkok, 10400 Thailand; 3 Department of Pathology, Faculty of Medicine, Ramathibodi Hospital, Mahidol University, Bangkok, Thailand; 4 Department of Surgery, Faculty of Medicine, Ramathibodi Hospital, Mahidol University, Bangkok, Thailand

**Keywords:** ddPCR, EIF2C1, FISH, HER2, IHC

## Abstract

Breast cancers with amplification and overexpression of human epithelial growth factor receptor 2 (HER2) are associated with poor prognosis, and targeted for anti-HER2 therapy. Immunohistochemistry (IHC) and fluorescence in situ hybridization (FISH) are currently the recommended methods to asses HER2 overexpression/amplification. Droplet digital PCR (ddPCR), a highly accurate method to quantify DNA copy number, is potentially a robust alternative for HER2 diagnostics. In the FISH assay and most of previous ddPCR reports, chromosome 17 centromere (CEP17) has been used as the reference control to determine HER2/CEP17 ratio. Nevertheless, miss-classification could occur when HER2 is co-amplified with CEP17. To avoid this inherent defect, in the present study, we employed ddPCR assay using the human eukaryotic translation initiation factor 2C1 (EIF2C1) gene located at chromosome 1p34.3 as the reference control to quantify HER2 copy number in 31 frozen breast cancer tissues. HER2 status of these samples had been determined by FISH and classified as HER2-amplified and HER2-non-amplified breast cancers. The results showed that HER2 determined by ddPCR using HER2/EIF2C1 ratio was in good concordance with HER2 determined by FISH using HER2/CEP17 ratio, the concordance rate 87.1% (27/31), Kappa  = 0.719. The sensitivity and specificity of ddPCR assay was 90% (9/10) and 85.7% (18/21), respectively. The median HER2/EIF2C1 copy number ratio in HER2-amplified cancers (6.55, range 1.3–17.3) was significantly higher than in HER2-non-amplified cancers (1.05, range 0.6–3.6, *p* < 0.001). This study demonstrated that ddPCR using HER2/EIF2C1 ratio could accurately assess HER2 status in frozen breast cancer tissues. Thus, our findings warrant further studies into breast cancer with HER2-equivocal by IHC/FISH.

## Introduction

Breast cancer is a heterogeneous disease and can be categorized into different subtypes based on expression of estrogen receptor (ER), progesterone receptor (PR), and human epidermal growth factor receptor-2 (HER2), which have different prognosis and treatment [1]. Amplification and overexpression of HER2/Neu are detected in approximately 25–30% of breast cancers and are strongly associated with poor prognosis [2, 3]. In addition, HER2 status has a therapeutic impact because monoclonal antibodies against HER2 (Trastuzumab, Pertuzumab, Trastuzumab emtansine) have been shown to be effective for treating HER2-positive breast cancer [4–9].

Immunohistochemistry (IHC) and fluorescence in situ hybridization (FISH) are currently the methods recommended by ASCO/CAP guidelines to asses HER2 status [10, 11]. Most laboratories initially investigate HER2 status with IHC which is easier to perform, but analysis of the results could be subjective and varied with different antibodies and observers. In contrast to IHC, FISH technique offers better diagnostic accuracy and added confidence, particularly when it is used to supplement weak IHC signals, but it is more labor intensive, time-consuming, and expensive.

Recently, droplet digital PCR (ddPCR) has been developed for absolute nucleic acid quantification [12]. Droplet digital PCR (ddPCR) is potentially an alternative technique to achieve a higher throughput capability and may yield a more accurate diagnosis for HER2 amplification. Concordance between HER2 status from ddPCR assays and HER2 status from standard HER2 assays has been reported [13–18]. In standard FISH assay, chromosome 17 centromere (CEP17) has been used as the reference control to determine HER2/CEP17 copy number ratio. However, misclassification of patients as HER2 non-amplified due to focal amplification of chromosome 17 centromere (CEP17) which leads to amplification of the reference region has been reported [19]. An alternative reference FISH probes have been suggested such as SMS, RARA, and TP53 genes to determine the true HER2 amplification status in patients with polysomy of chromosome 17 [19]. Nevertheless, most of the previous studies on detection of HER2 amplification by ddPCR still used CEP17 region as the reference control [13, 15–18]. Only one study used elongation factor Tu GTP binding domain containing 2 (EFTUD2) gene located at 17q21.31 as an alternative chromosome 17 probe [14].

It might be worthy to seek for other reference probes that can identify true HER2 amplification, especially in CEP17 co-amplification cases. The human eukaryotic translation initiation factor 2C1 (EIF2C1) gene located at 1p34.3 has been suggested by Bio-Rad as a reference control for HER2 copy number quantification. However, HER2 status measurement in breast cancer determined by ddPCR using EIF2C1 as the reference control has not been reported before. Thus, in this study, we sought to quantify HER2 amplification in breast cancer tissues by employing ddPCR assay using EIF2C as the reference control and compared our results with those obtained by FISH assay for the same samples. HER2 status determined by FISH was considered according to the 2013 ASCO/CAP HER2 guideline, and HER2/CEP17 ratio ≥ 2 was considered as HER2 amplification [11].

## Materials and methods

### DNA extraction

Breast cancer tissues have been obtained from Ramathibodi Hospital and kept at − 80 °C. HER2 status of these samples had been determined by FISH/or IHC in our previous report [20]. The study protocol was approved by the Ethics Committee of the Faculty of Medicine, Ramathibodi Hospital, Mahidol University.

DNA of 31 samples snap frozen breast cancer tissues which have been confirmed by an experienced breast pathologist were isolated with QIAamp DNA Tissue Kit (Qiagen, Valencia, CA, USA) according to the manufacturer’s instructions and the quantity was measured by Nanodrop.

### Digital PCR

Digital PCR was performed using the droplet digital PCR (ddPCR) method on Bio-Rad QX200™ (Bio-Rad, Hercules, CA, USA). A total 20 µl PCR reaction was prepared with 15–20 ng DNA and 2X ddPCR Supermix for probe (Bio-Rad, Hercules, CA, USA); primers and fluorescent probes (FAM and VIC) were prepared from Prime PCR assay for ddPCR (dHsaCP1000116 for HER2 and dHsaCP2500349 for EIF2C1 as the reference control). HindIII was mixed in PCR reaction. Droplets were generated by Bio-Rad QX200 droplet generator. Then, the total 40 µl of emulsified PCR reactions were transferred to a 96-well plate and heat sealed before running on T1000 thermal cycler (Bio-Rad, Hercules, CA, USA) with the following cycle: 95 °C for 10 min, 40 cycles of 94 °C for 30 s and 60 °C for 60 s, 98 °C for 10 min, and hold at 4 °C. The temperature ramp rate was 2°C/s for all steps. Negative control with no DNA was included in each run. After the PCR, the PCR plates were transferred to Bio-Rad QX200 droplet reader. Analysis of ddPCR data was performed by using QuantaSoft v1.3.2.0 software from Bio-Rad. HER2/EIF2C1 ratio ≥ 2.0 was defined as HER2 amplification, and HER2/EIF2C1 ratio < 2.0 was defined as HER2 non-amplification.

### Fluorescence in situ hybridization (FISH)

FISH analysis was performed in isolated nuclei using the PathVysion HER2 DNA probe kit (Vysis, Illinois, USA) following the manufacturer’s protocol as previously published [20]. As proposed by the ASCO/CAP guideline [11], HER2/CEP17 ratio < 2 was considered HER2 negative, and HER2/CEP17 ratio ≥ 2 was considered HER2 positive.

### Immunohistochemistry (IHC)

IHC for HER2 overexpression was performed according to standard methods as previously described [20] and scored according to the ASCO/CAP guideline as negative (0, 1 +), equivocal (2 +), or positive (3 +) [11].

### Statistical analysis

Statistical analyses were performed with SPSS v.11.5 (SPSS Inc., Chicago, Illinois, USA) or GraphPad Prism 7 (version 7.03). Kappa coefficient was used to determine the concordance between FISH and ddPCR method in detecting HER2 amplification.

## Results

**Fig. 1:**
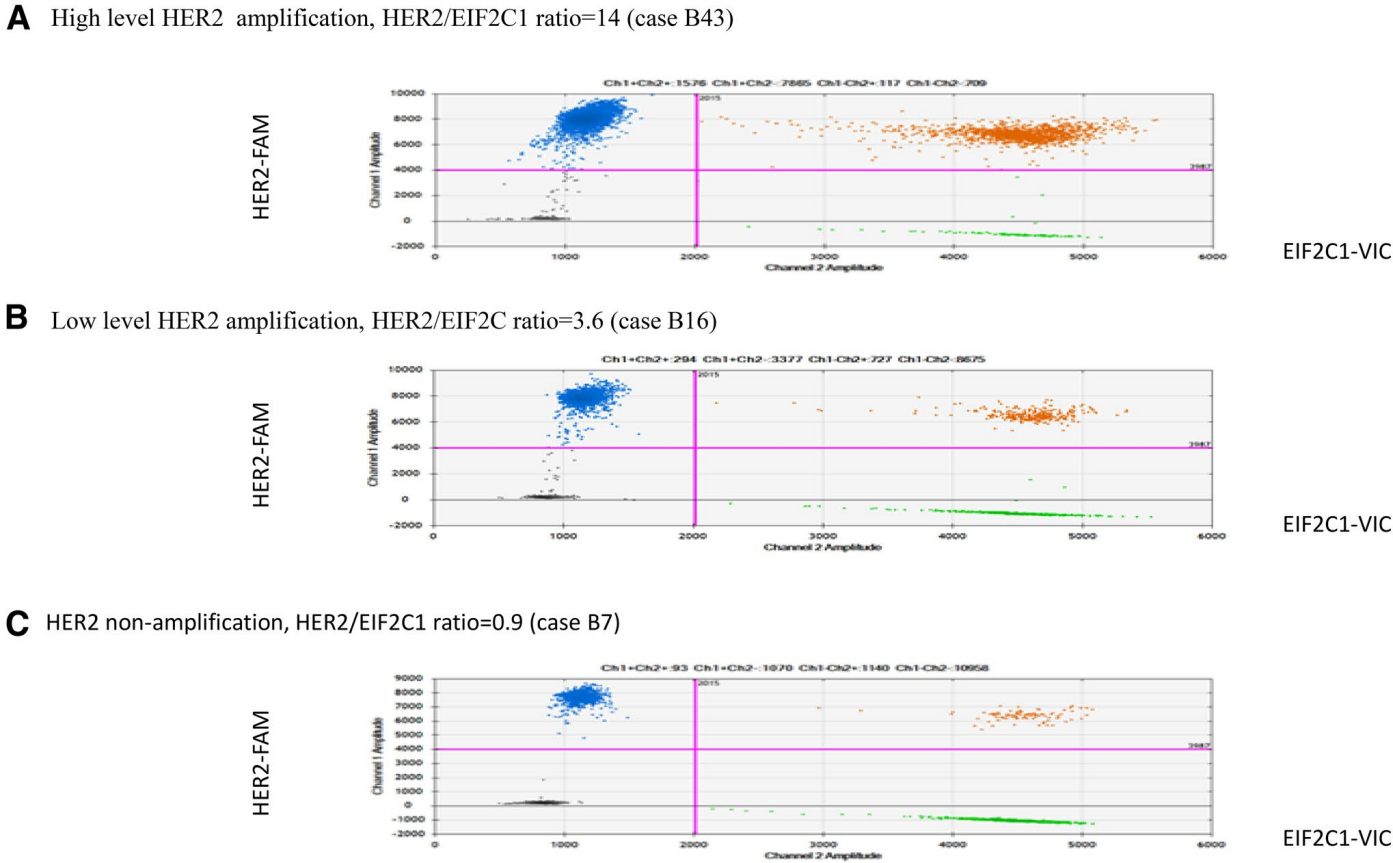
Representative of HER2 amplification detected by ddPCR in breast cancer tissues using EIF2C1 as the reference control. HER2 and EIF2C1 were labeled with FAM and VIC fluorescent probe, respectively. **a** High-level HER2 amplification, **b** low-level HER2 amplification, **c** HER2 non-amplification. In each subfigure, the four quadrants represent top left: droplets with HER2 DNA only, top right: droplets with both HER2 and EIF2C1 DNA, bottom right: droplets with EIF2C1 DNA only, and bottom left: droplets with no DNA

**Fig. 2:**
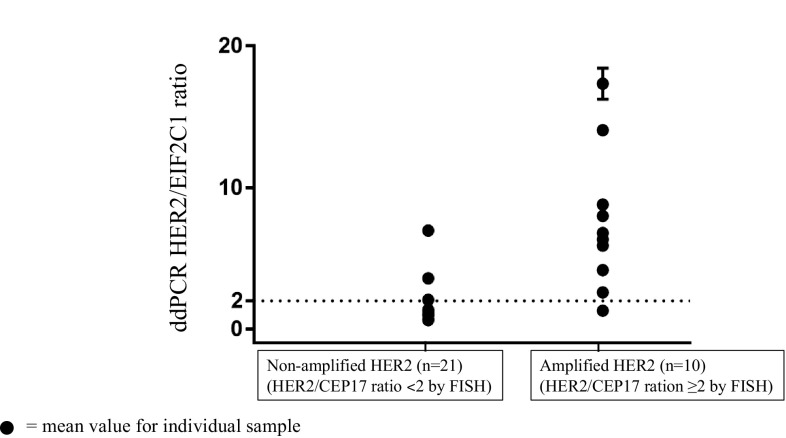
Reproducibility of HER2/EIF2C1 ddPCR running in triplicate for individual sample from HER2-negative (non-amplified) and HER2-positive (amplified) breast cancer tissues detected by FISH. Mean ± SD for HER2/EIF2C1 ratio is shown for individual sample. The error bar is shorter than the mean symbol in most of the samples

**Fig. 3:**
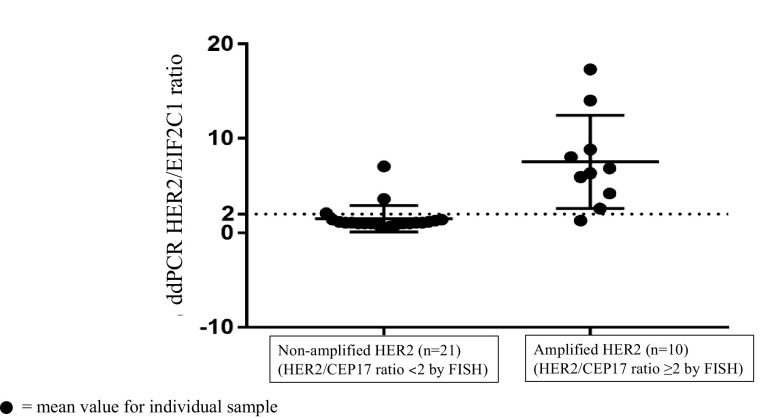
HER2/EIF2C1 ratio was assessed by ddPCR on DNA from HER2-negative (non-amplified) and HER2-positive (amplified) breast cancer tissues detected by FISH. The mean values of HER2/EIF2C1 ratio for individual sample and the mean ± SD error bar for the groups are shown

In this study, HER2 amplification was determined in 31 frozen breast cancer tissues by ddPCR. HER2 status of these samples had been determined by FISH/or IHC in the previous report [20]. Of the 31 breast cancer tissues, HER2 status was defined by FISH as HER2-amplified and HER2-non-amplified breast cancers. Clinicopathologic characteristics of patients with invasive breast carcinomas are shown in Table 1. We assessed the potential of ddPCR using HER2/EIF2C1 copy number ratio for detection of HER2 amplification. HER2/EIF2C1 copy number ratio in each sample was determined in three different experiments. Representative of ddPCR plots are shown in Fig. 1. Data of HER2 status determined by IHC, FISH, and ddPCR are shown in Table 2. The reproducibility of three replicates was tight as shown in Fig. 2. The threshold for the HER2/EIF2C1 copy number ratio ≥ 2.0 was defined as HER2 amplification, consistently with ASCO/CAP guideline for HER2/CEP17 ratio [11]. The median HER2/EIF2C1copy number ratio in HER2-amplified breast cancers (6.55, range 1.3–17.3) was significantly higher than in HER2-non-amplified breast cancers (1.05, range 0.6–3.6, *p* = 0.000 Mann–Whitney *U* test), as shown in Fig. 3. HER2 status determined by ddPCR using HER2/EIF2C1 ratio was in good concordance with HER2 status determined by FISH using HER2/CEP17 ratio, the concordance rate 87.1% (27/31), Kappa = 0.719. The sensitivity and specificity of ddPCR assay was 90% (9/10) and 85.7% (18/21), respectively, as shown in Table 3.

**Table 1:** Clinicopathologic characteristics of patients with invasive breast carcinomas (*n* = 31)

Characteristics	Number of patients	%
Age (years)		
< 50	16	51.6
> 50	15	48.4
Tumor grade		
1–2	18	58
3	13	42
Lymph node status		
Negative	12	38.7
Positive	19	61.3
Estrogen receptor status		
Negative	20	64.5
Positive	11	35.5
HER2 status		
Negative	21	64.5
Positive	10	35.5

**Table 2:** HER2 status determined by IHC, FISH, and ddPCR

Case no.	IHC	HER2/CEP17 ratio (FISH)	HER2/EIF2C1 ratio Triplicate (ddPCR)			HER2/EIF2C1 ratio Mean ± SD (ddPCR)
B1	ND	Amp	9.0	8.6	8.8	8.8 ± 0.20
B2	ND	1.0	1.0	1.0	0.9	1.0 ± 0.06
B4	3 +	Amp	8.4	7.9	7.7	8.0 ± 0.36
B5	3 +	0.8	0.9	0.9	1.2	1.0 ± 0.17
B7	ND	1.1	0.9	0.9	0.9	0.9 ± 0.00
B8	1 +	1.1	1.0	1.0	0.9	1.0 ± 0.06
B9	ND	1.8	0.6	0.6	0.7	0.6 ± 0.06
B11	3 +	Amp	6.1	6.0	5.6	5.9 ± 0.26
B13	1 +	1.1	1.3	1.3	1.2	1.3 ± 0.06
B14	3 +	Amp	4.2	4.1	4.2	4.2 ± 0.06
B15	2 +	1.1	1.2	1.2	1.0	1.1 ± 0.12
B16	2 +	1.1	3.6	3.6	3.6	3.6 ± 0.00
B18	1 +	1.0	1.1	1.1	1.1	1.1 ± 0.00
B19	3 +	Amp	18.5	16.3	17.2	17.3 ± 1.11
B21	0	1.9	1.4	1.3	1.4	1.4 ± 0.06
B23	1 +	1.0	1.0	1.0	1.1	1.0 ± 0.06
B24	2 +	1.0	1.9	2.0	2.3	2.1 ± 0.21
B25	ND	1.7	1.1	1.2	1.2	1.2 ± 0.06
B26	ND	1.5	1.3	1.3	1.5	1.4 ± 0.12
B28	3 +	Amp	6.9	6.6	6.9	6.8 ± 0.17
B29	ND	Amp	6.3	6.5	6.2	6.3 ± 0.15
B32	ND	Amp	2.7	2.5	2.6	2.6 ± 0.10
B35	ND	1.1	1.0	0.9	0.9	0.9 ± 0.06
B36	ND	1.1	1.0	1.0	1.0	1.0 ± 0.00
B37	ND	Amp	1.3	1.3	1.3	1.3 ± 0.00
B38	ND	1.6	1.1	1.05	1.1	1.1 ± 0.30
B40	ND	1.0	0.7	0.7	0.7	0.7 ± 0.00
B41	ND	1.0	1.3	1.2	1.2	1.2 ± 0.06
B43	3 +	Amp	14.1	13.8	14.2	14.0 ± 0.21
B48	ND	1.0	6.7	7.2	7.0	7.0 ± 0.25
B62	1 +	1.0	1.1	1.1	1.1	1.1 ± 0.00

*Amp* HER2 amplification as cluster, *ND* not done

**Table 3:** HER2 status in breast cancers tissues assessed by ddPCR and FISH (*n* = 31)

		HER2/CEP17 (FISH)	
		Negative	Positive
HER2/EIF2C1 (ddPCR)	Negative	18 (85.7%)	1 (10%)
	Positive	3 (14.3%)	9 (90%)

The sensitivity and specificity of ddPCR assay; 90% (9/10) and 85.7% (18/21), respectively

## Discussion

Due to the prognostic and therapeutic impacts, correct identification of patients with HER2 amplification is crucial. There are several methods to determine HER2 status. However, FISH and IHC are currently the methods recommended by ASCO/CAP guidelines [10, 11]. Most laboratories initially investigate HER2 status with IHC and the result scored as 1 + (negative), 2 + (equivocal), and 3 + (positive). IHC analysis is prone to the subjective evaluation of images and the results can be varied with different antibodies and observers. FISH analysis usually uses a probe to the HER2 gene and another for CEP17, as the reference control. An HER2/CEP17 ratio of less than 1.8 is considered negative, 1.8–2.0 is considered equivocal, and more than 2.0 is considered positive for HER2 amplification, as the ASCO/CAP guidelines [11]. FISH is more labor intensive, time-consuming, and expensive method. In addition, several studies [19, 21–23] have reported that true polysomy of chromosome 17 is rare. Patients with increased HER2 copy numbers along with increased CEP17 copy numbers might be misclassified as non-amplified HER2. In principle, ddPCR and FISH methods are both based on nucleic acid level. Absolute HER2 copy number can be determined by ddPCR without the need for calibration curves. This technique may be useful as an alternative to IHC/FISH. The advantage of this method is that it enables objective evaluation, robustness, and high throughput.

In this study, the concordance rate for HER2 detection by ddPCR and FISH was 87.1% (27/31), with 3 samples (B16, B24, and B48) that were classified as HER2 amplified by ddPCR but HER2 non-amplified by FISH, as shown in Tables 2 and 3. There was only one sample (B37) which was classified as HER2 non-amplified (ratio = 1.3) by ddPCR but HER2 amplified (uncountable) by FISH. CEP17 did not gain in both B16 and B48, and IHC showed 2 + in B16; therefore, the discordance in these samples might not be due to CEP17 gain. For B24, CEP17 gained, IHC showed 2 + , and ddPCR showed low-level amplification (ratio = 2.1). The discordance of HER2 status in B37 sample might be due to intratumoral heterogeneity of the sample. Copy number analysis by ddPCR is based on an average of all cell DNA in the sample. Therefore, to maintain sensitivity in the sample which is contaminated with high normal DNA, the lower ratio should be applied [14]. Ten samples with high-level HER2 amplified by FISH showed HER2 copy amplification as a cluster, suggesting that the amplification of these samples was homogeneous staining region type (HSR). Of these samples, FISH could not detect accurate copy number due to uncountable positive signals while accurate copy number could be achieved by ddPCR. Of the 10 samples with high-level HER2 amplification by FISH, CEP17 gain occurred in 6 samples. From these results, we observed that CEP17 gain did not affect HER2 classification in the samples with high-level HER2 amplification by FISH. The good concordance between HER2 amplification by ddPCR and HER2 amplification by FISH in breast cancer has been reported [13–17]. Among these prior studies, most of them used CEP17 region as the reference control [13, 15–17]; only one study used EFTUD2 as an alternative chromosome 17 probes [14].

This study demonstrated that ddPCR using HER2/EIF2C1 ratio could accurately assess HER2 status in frozen breast cancer tissues. Thus, our findings warrant further studies to examine whether ddPCR using HER2/EIF2C1 ratio could discriminate the breast cancer with HER2-equivocal by IHC/FISH.
